# Supplementary material for: Tracking Thermal Transport in Colloidal Quantum Dot Films Using in Situ Time-Resolved X‑ray Diffraction
Source: Nano Lett. 2026 Jul 30;26(32):10725–33. doi: 10.1021/acs.nanolett.6c02198 (PMC13495939; doi:10.1021/acs.nanolett.6c02198)
Supplement: Supplementary file 1 [file nl6c02198_si_001.pdf]

Supporting Information for Tracking thermal transport in colloidal quantum dot films using *in-situ* time-resolved X-ray diffraction

Eliza Wieman[1], Nejc Nagelj[2], Ethan Curling[3], Larry Chen[4], Jin Yu[5], A. Paul Alivisatos[3,6], Aaron M. Lindenberg[7,8], Benjamin T. Diroll[9], Jacob H. Olshanky[2], Jihong Ma[10]*, Burak Guzelturk[5]*, Benjamin L. Cotts[1]*

1. Department of Chemistry, Middlebury College, Middlebury, VT, 05753, USA
2. Department of Chemistry, Amherst College, Amherst, MA, 01002, USA
3. Department of Chemistry, University of California at Berkeley, Berkeley, CA, 94720, USA
4. Department of Materials Science and Engineering, Northwestern University, Evanston, IL, 60208, USA
5. X-ray Science Division, Argonne National Laboratory, Lemont, IL, 60439, USA
6. Department of Chemistry, University of Chicago, Chicago, IL, 60637, USA
7. Department of Materials Science and Engineering, Stanford University, Stanford, CA, 94305, USA
8. SLAC National Accelerator Laboratory, Menlo Park, CA 94025, USA
9. Center for Nanoscale Materials, Argonne National Laboratory, Lemont, IL, 60439, USA
10. Department of Mechanical Engineering, University of Vermont, Burlington, VT, 05405, USA

correspondence to: jihong.ma@uvm.edu, burakg@anl.gov, bcotts@middlebury.edu

Contents:

A. Synthesis and sample preparation
B. Atomic Force Microscopy measurement of thin-film film for film thickness
C. TR-XRD data collection and analysis
D. Differential signal example for liquid jet data
E. Calculation of excitation profile and expected ΔT
F. ASE Measurement Methods
G. Estimation of thin-film packing density and heat capacity
H. Analytical model and FEM model fitting of recovery data
I. Application of FEM model to approximate CW heating

A. Synthesis and sample preparation

Solid state CdSe:CdS quantum dot (QD) thin-film sample

Thin-film CdSe:CdS QD data were collected from an 8-monolayer CdS shell sample from the same batch as detailed in Ref[1,2]. As previously described, the CdSe cores were prepared via hot injection synthesis, yielding a 3.5 nm diameter from optical sizing. The total average size of the core-shell QD was measured to be 9.4 nm by TEM. Full details can be found in the supporting information of Ref[2].

The stock solution in hexanes was washed via centrifugation to remove excess ligands using methyl acetate as an antisolvent. The supernatant was resuspended in toluene and a 600 nm (see below for thickness measurement) film was dropcasted onto a fused silica substrate for measurement at APS.

Liquid CdSe:CdS sample

Liquid jet data was collected over two beamtimes at APS on two similar samples. Measurements were conducted on the same batch as the thin-film sample resuspended in dodecane, a higher boiling point solvent for greater stability during measurement. Additional measurements were conducted on a second sample that was 11.6 nm in size by TEM sizing with consistent results.

B. Atomic Force Microscopy measurement of thin-film film for film thickness

Atomic force microscopy images were taken of the thin-film CdSe:CdS films at the Center for Nanoscale Materials after measurements were complete at Argonne Advanced Photon Source using a Veeco Multimode 8 SPM in tapping mode. The sample was scratched to expose the bare substrate as a reference height. Multiple images were then taken along the scratch to determine the average sample height. In the right panel of Figure S1, an average line profile is shown with the bare substrate from the scratch on the right representing the zero height. On average, the film was 606 ± 68 nm. This was rounded to 600 nm in simulations.

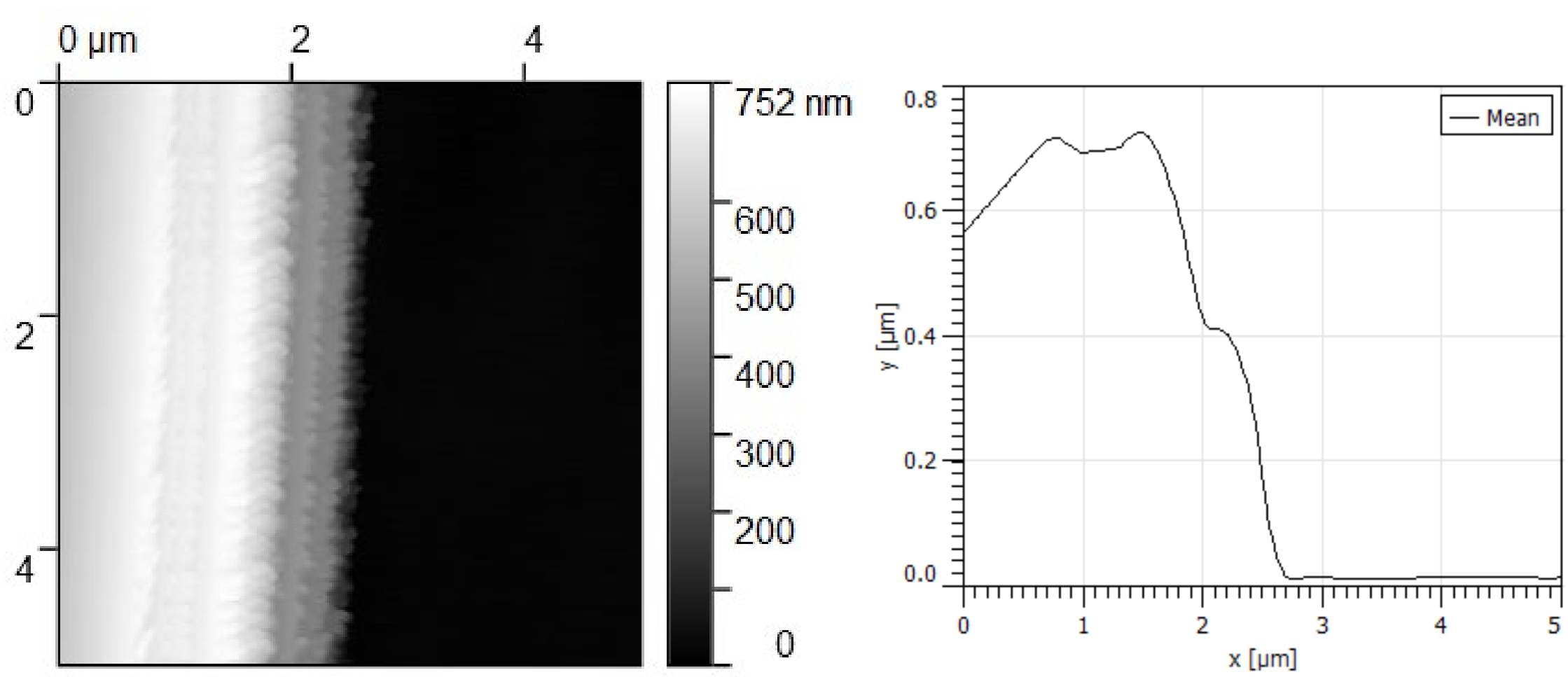

Figure S1: Representative tapping-mode AFM image of scratch in thin-film CdSe:CdS film following measurement at APS 11-ID-D. Line profile is averaged over all rows of the image. Multiple images were taken and on average the film was 606 nm thick.

C. TR-XRD data collection and analysis

TR-XRD measurements were performed at the Beamline 11-ID-D of the Advanced Photon Source at the Argonne National Laboratory. Liquid jet experiments were conducted in a transmission geometry and thin-film experiments were conducted in a grazing incidence geometry (see Figure 1). For all experiments a frequency doubled Ti:Sapphire laser (10 kHz repetition, 1.2 ps pulse width) was used to provide a 3.1 eV ultrafast pump beam. X-ray scattering signal was collected with a Pilatus 2M detector with 300 μm thick silicon active area and pixel size of 172 by 172 μm. The Pilatus detector was electronically gated at the repetition rate of the laser to collect pump-probe signal. Temporal overlap was obtained using a fast rise time photodiode. Spatial overlap was checked with a 300 μm pinhole at the sample location. The pump-probe delay was controlled electronically by a phase shifter (Colby Instruments) and an electronic delay generator (Highland V85x). Delay points were randomized in each experiment to minimize systematic errors and repeated over multiple iterations to increase signal to noise via averaging.

Thin-film thin-film TR-XRD data collection

Thin-film TR-XRD was collected at the same beamline in a grazing incidence geometry. The same laser system was used. The X-ray probe was 11.7 keV (50 ps, 78 MHz, 324 bunch mode) with a beam size of 15 μm by 450 μm. We used a grazing incidence of about 0.3 degrees. To match the elongated stripe like shape of the X-ray probe beam on the sample surface, we used a cylindrical lens to perform one dimensional focusing of the 400 nm laser beam, overfilling the X-ray beam by a factor of 2. A CeO_2 sample was used to calibrate the sample-to-detector distance (22.72 cm). The maximum measured Q range was 5.36 Å^{-1}. The laser fluence was 0.7 mJ/cm^2 at 3.1 eV. An acquisition time of 12 seconds was used for each delay point.

Liquid jet TR-XRD data collection

For liquid data, the sample was diluted into dodecane and recirculated through a round liquid jet with a diameter of 700 μm at the measurement location. The X-ray probe beam was 16.5 keV with an 80 ps pulse width and 6.5 MHz repetition rate (APS 24-bunch mode). The size of the X-ray probe was 60 μm (V) × 450 μm (H) on the sample location. A CeO_2 sample was used to calibrate the sample-to- detector distance. For the first beamline experiment, used to collect the liquid time-dependent recovery data shown in Figure 4, a sample-to-detector distance of 17.3 cm was used. This resulted in a maximum measured Q range of 7 Å^{-1}, partially limited by sample holder geometry. For each delay point, diffraction data was acquired for 60 sec. For the second beamline experiment, used for the DWF data shown in Figure S3, a sample-to-detector distance of 16.8 cm was used for a maximum measured Q range of 8.5 Å^{-1}. For each delay point, diffraction data was acquired for 14 sec.

TR-XRD data analysis

QXRD software was used to convert scattering images to a diffraction pattern vs Q. All further data processing was conducted with Matlab. Background subtracted images were sorted by pump-probe delay and averaged to get I(Q, t) at each time point. All images collected before t = 0 were averaged together to calculate the baseline signal I_0. ΔI(Q,t) was then calculated by subtracting the baseline from each averaged time point ($\Delta I(Q,t)= I(Q, t)- I_0$). Data of this form is presented in Figure 3a and Figure 4. The signal was examined for consistency across all major lattice peaks. A table listing the major peaks and their corresponding lattice-plane assignments is included below.

Table S1: Reciprocal lattice planes cited in the main text and their corresponding Q values. Planes were assigned to peaks using ICDD reference tables (PDFs 000-041-1049 and 00-008-0459)[3]. **Bold** planes were resolvable in both the liquid-jet and thin-film data, whereas the differential signal for non-bold planes was only clearly resolved in the thin-film diffraction patterns.

Table S1. Assignments of major X-ray diffraction reflections for CdSe/CdS QDs

Lattice Plane	Q ($Å^{-1}$)
**(100)**	1.75
**(002)**	1.86
**(101)**	1.98
(102)	2.55
**(110)**	3.03
**(103)**	3.30
**(112)**	3.55
(201)	3.72
(202)	3.97
**(203)**	4.46
(210)	4.63
**(211)**	4.71
(114)	4.79

TR-XRD DWF thermometry

Transient DWF thermometry transforms the data to extract lattice temperature of the nanocrystals as a function of time. Full details can be found in Ref[1]. Prominent peaks with high differential signal-to-noise were identified in background-subtracted diffraction patterns. The time-dependent intensity I(Q, t), was calculated for each peak by averaging intensities over a total width of 0.0268 $Å^{-1}$ around the selected Q. Then $-\ln(I/I_0)$ vs Q^2 is plotted for each peak and fit with a line to extract the slope, as seen for both liquid jet and thin-film data in Figure 3b/c. From the following equation for Debye-Waller Factor[1]

$$-\ln\left(\frac{I(t)}{I_0}\right) = \frac{Q^2}{3}\left\langle\left(\Delta u(t)\right)^2\right\rangle \quad (1)$$

we see that the slope is equal to $\frac{\left\langle\left(\Delta u(t)\right)^2\right\rangle}{3}$. Using the following relationship specific for CdSe:CdS (described in SI of Ref[1])

$$\left\langle\left(\Delta u(t)\right)^2\right\rangle = 0.000223\ Å/\mathrm{K} * \Delta T\ , \quad (2)$$

ΔT can then be extracted from the slope of the DWF plots by

$$\Delta T = \frac{3*slope}{0.0002223} \quad (3)$$

Equation 3 was used to determine temperature change observed in the TR-XRD experiments at selected time points.

TR-XRD transient peak-shift thermometry

To demonstrate the fidelity of the transient DWF analysis, we perform a second, independent approach for transducing the differential TR-XRD signal into time-dependent temperature changes. Specifically, we use the volumetric thermal expansion coefficient to convert reflection peak shifts into $\Delta T(\tau_d)$. For this analysis, the value of α=2.3 x 10^{-5} K^{-1} is applied to all reflections. This effective

volumetric thermal expansion coefficient value was taken from Badlyan et. al. who derived/measured it for CdSe/CdS quantum dots using temperature-dependent Raman spectroscopy.[4]

We fit Bragg peak positions using a pseudo-Voight function on background-subtracted data locally in the Q domain, then extract the peak Q value as a function of time. Below we show the results from the <103> wurtzite peak. Figure S2 shows the strain ($\Delta Q/Q_0$) and absolute value of Q as a function of time delay. As the peaks shift to lower Q, the strain indicates transient lattice expansion, as expected from thermal heating.

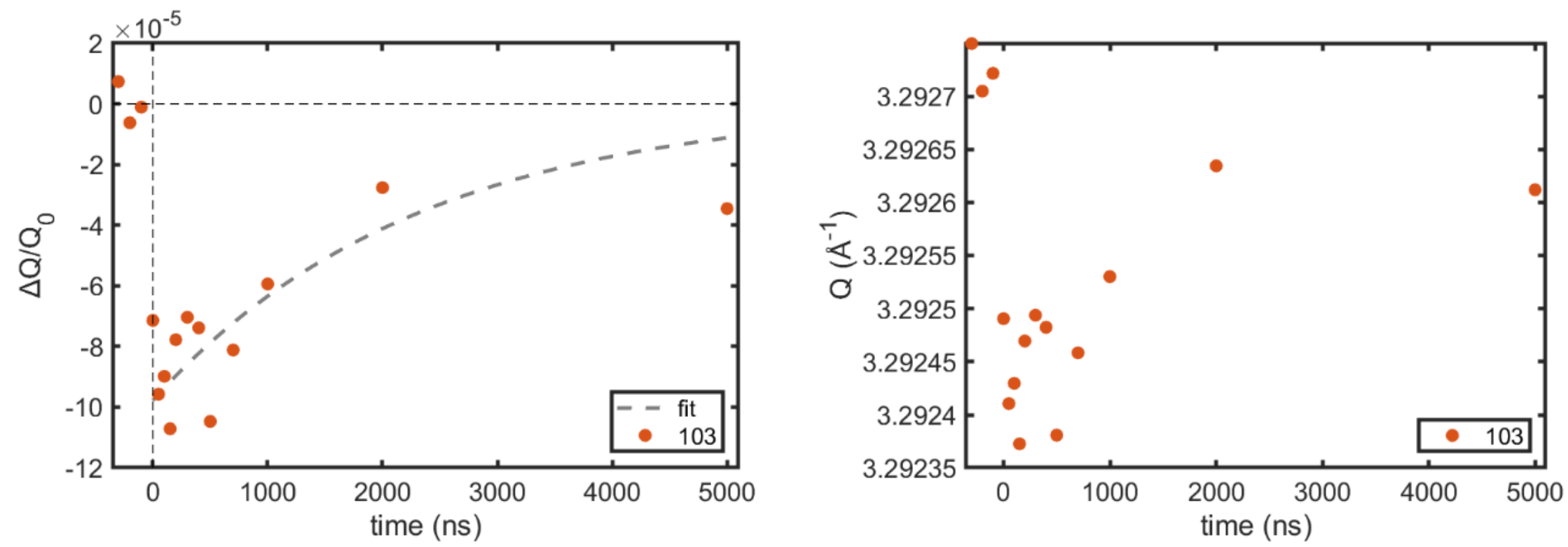

Figure S2: Transient peak shift analysis for the <103> reflection of the thin-film sample. The change in Q over the initial Q, $\frac{\Delta Q}{Q_0}$, as a function of time delay for the thin-film sample's is shown on the left, with an exponential fit shown in dashed line. The absolute Q value of the peak is shown on the right.

From the Bragg peak position shifts, we extract both the maximum temperature jump at selected time points, 50 and 5000 ns, and the overall relaxation time scale. We find the ΔT at 50 ns falls within a 4.3-5.9 K range, consistent with the 5.5 K obtained from the Debye-Waller analysis (Table R3). Similarly, the ΔT at 5000 ns falls within a 1.3-2.5 K range, consistent with the 2.0 K measured from Debye-Waller analysis (Table R4). These particles have a photoluminescence lifetime of 42 ns,[2] so the agreement at late times, dominated by the thermal signature, is particularly encouraging, as the Reviewer suggested. In addition, we fit the strain recovery lifetime to a single exponential and obtain a lifetime 2.3 ± 0.8 μs, consistent with the 2.37 ± 0.34 μs from the DWF analysis. The larger uncertainty likely reflects the greater susceptibility of peak-shift measurements to background scattering noise compared to peak intensity changes.

Table S2. Peak-Shift Transient Thermal-Expansion Analysis for the Thin-Film Sample at 50 ns.

**hkl**	**Q_i (Å^{-1})**	**ΔQ (Å^{-1})** τ=50 ns	**ΔT (K)**
"100"	1.75357	-0.00025	6.2
"002"	1.86375	-0.00021	5.0
"101"	1.97963	-0.00024	5.4
"102"	2.55564	-0.00024	4.1
"110"	3.02734	-0.00040	5.8
"103"	3.29272	-0.00032	4.2
"112"	3.55587	-0.00044	5.4
		**Average ΔT**	5.1

StDev	0.8

Table S3. Peak-Shift Transient Thermal-Expansion Analysis for the Thin-Film Sample at 5000 ns.

**hkl**	**Q_i** (Å^{-1})	**ΔQ** (Å^{-1}) τ=5000 ns	**ΔT**
"100"	1.75357	-0.00005	1.1
"002"	1.86375	-0.00013	3.0
"101"	1.97963	-0.00010	2.2
"102"	2.55564	-0.00011	1.9
"110"	3.02734	-0.00013	1.9
"103"	3.29272	-0.00011	1.5
"112"	3.55587	-0.00014	1.7
		**Average ΔT**	1.9
		**StDev**	0.6

The peak shift analysis returns an average ΔT of 1.9 ± 0.6 K at 5000 ns. A simple DWF fit returns a value of 1.96 K for the ΔT at the same time point, whereas an error weighted linear fit returns a ΔT of 2.3 ± 0.2 K. This near agreement increases our confidence in the DWF analysis. The greater noise in the liquid jet data, particularly background fluctuations from jet instability, precludes successful application of this peak shift approach.

D. Differential signal example for liquid jet data

A reference background-subtracted diffraction pattern and the $\Delta I(Q, t = 50$ ps) signal are shown below. The decrease in peak intensity is consistent with a transient Debye–Waller effect arising from lattice heating.

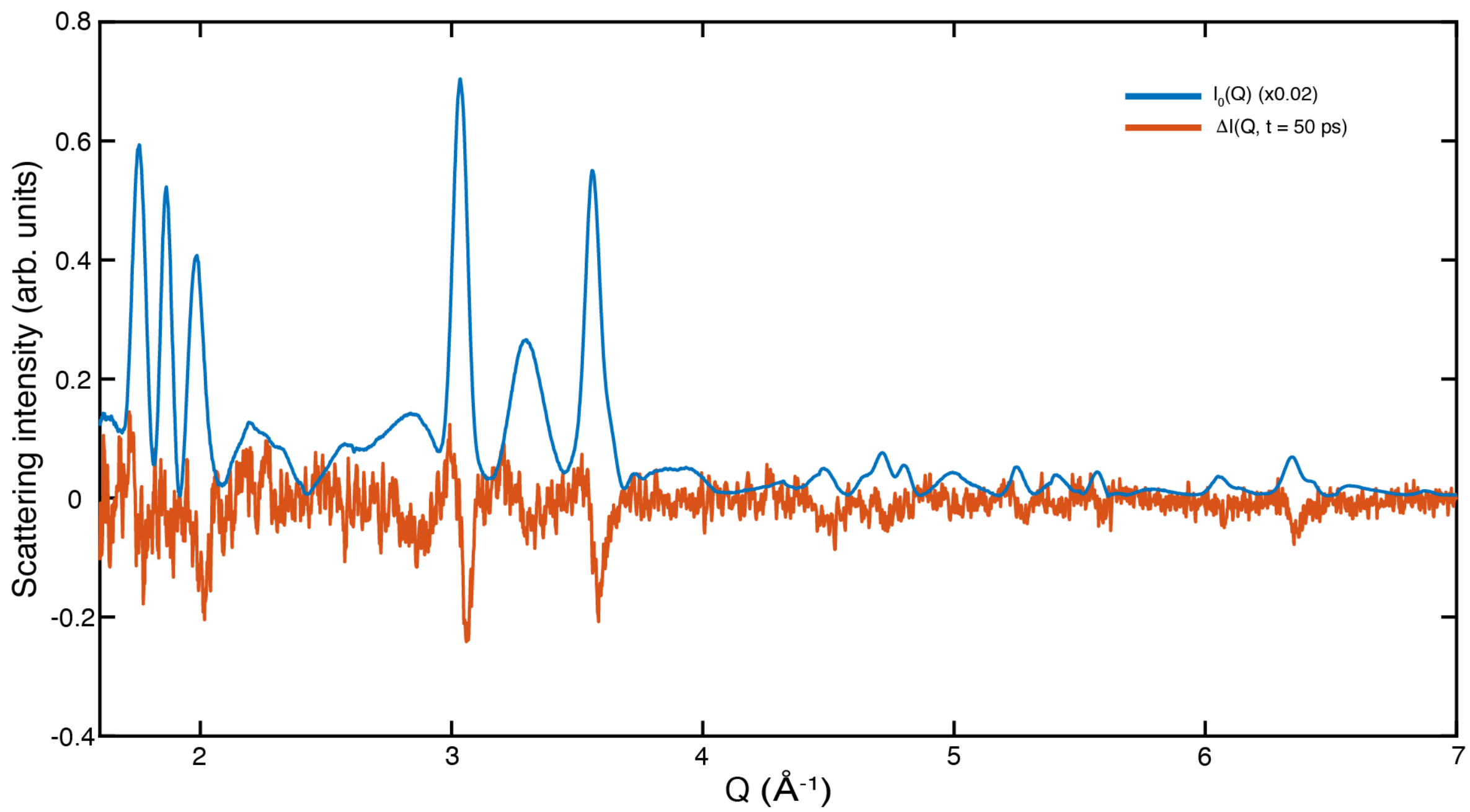

Figure S3: The liquid-jet differential signal clearly indicates lattice heating. The background-subtracted baseline I_0 signal (blue) is scaled to 2% of its actual intensity to appear on the same scale as the $\Delta I(Q,t)$ signal. The liquid-jet $\Delta I(Q,t)$ in orange shows decreased peak intensity, consistent with Debye–Waller lattice heating.

E. Calculation of excitation profile and expected ΔT

In grazing-incidence TR-XRD, the probe interrogates a shallow region, so the measured scattering volume lies largely perpendicular to the pump beam propagation direction, yielding improved effective pump–probe overlap. By contrast, in concentrated liquid jets the pump and probe are nearly collinear, and the pump fluence decays along the depth/optical path length, reducing the fraction of the probed volume that is uniformly excited.

To illustrate the greater uniformity of the differential signal in the grazing incidence thin-film measurement compared with the transmission liquid jet experiment, we estimate the expected number of excitations per dot, $\langle N \rangle$, and the resulting ΔT across the penetration depth of the film and the liquid jet. This simulation considers only the initial energy deposited by photoexcitation and neglects thermal diffusion.

Estimating expected ΔT for a given laser fluence

To estimate the temperature increase, ΔT, of each nanocrystal from laser heating for each experiment, the expected number of excitations per dot, $\langle N \rangle$, was calculated from the laser fluence, extinction coefficient (σ), and sample concentration.

$$\langle N \rangle = \frac{\sigma(cm^2) \cdot fluence\left(\frac{J}{cm^2}\right)}{E_{photon}(J)}$$

Then, $\langle N \rangle$ was converted to ΔE using:

$$\Delta E = (\langle N \rangle - 1)E_{photon} + E_{photon} - bandgap_{QD}$$

assuming that all but one excitation undergo Auger recombination to release all their energy to the QD lattice, whereas the final excitation undergoes fluorescence.

Subsequently, ΔE was converted to ΔT using:

$$\Delta T = \frac{\Delta E}{C_{v(QD)}}$$

where $C_{v(QD)}$ was calculated as a volume-weighted average of heat capacity of CdSe and CdS.

To simulate contributions to differential signal, the ΔT was discretized stepwise across each of the samples. ΔT was calculated at several jet/film depths, with linear absorption of the laser assumed throughout the sample. For liquid jet calculations, the 700-micron jet was split into 350 steps of 2 microns each. The 600 nm thin-film film was split into 60 steps of 10 nm. Absorbance of one step was calculated using Beer's Law and sample concentration, determined via UV-Vis. Absorbance values were then converted into percent transmittance (%T). Starting at the front of the jet (liquid) or sample surface (thin-film), $\langle N \rangle$ was calculated at each step with the experimental fluence used initially and then recalculated at each step by multiplying by %T.

Figures S4 and S5 show $\langle N \rangle$ and ΔT as a function of film/jet depth in thin-film and liquid samples, respectively. The decay in $\langle N \rangle$ and ΔT is due to absorption of the laser as it penetrates the sample. Expected $\langle N \rangle$ and ΔT values for each experiment were determined by averaging over the calculated values at each jet depth.

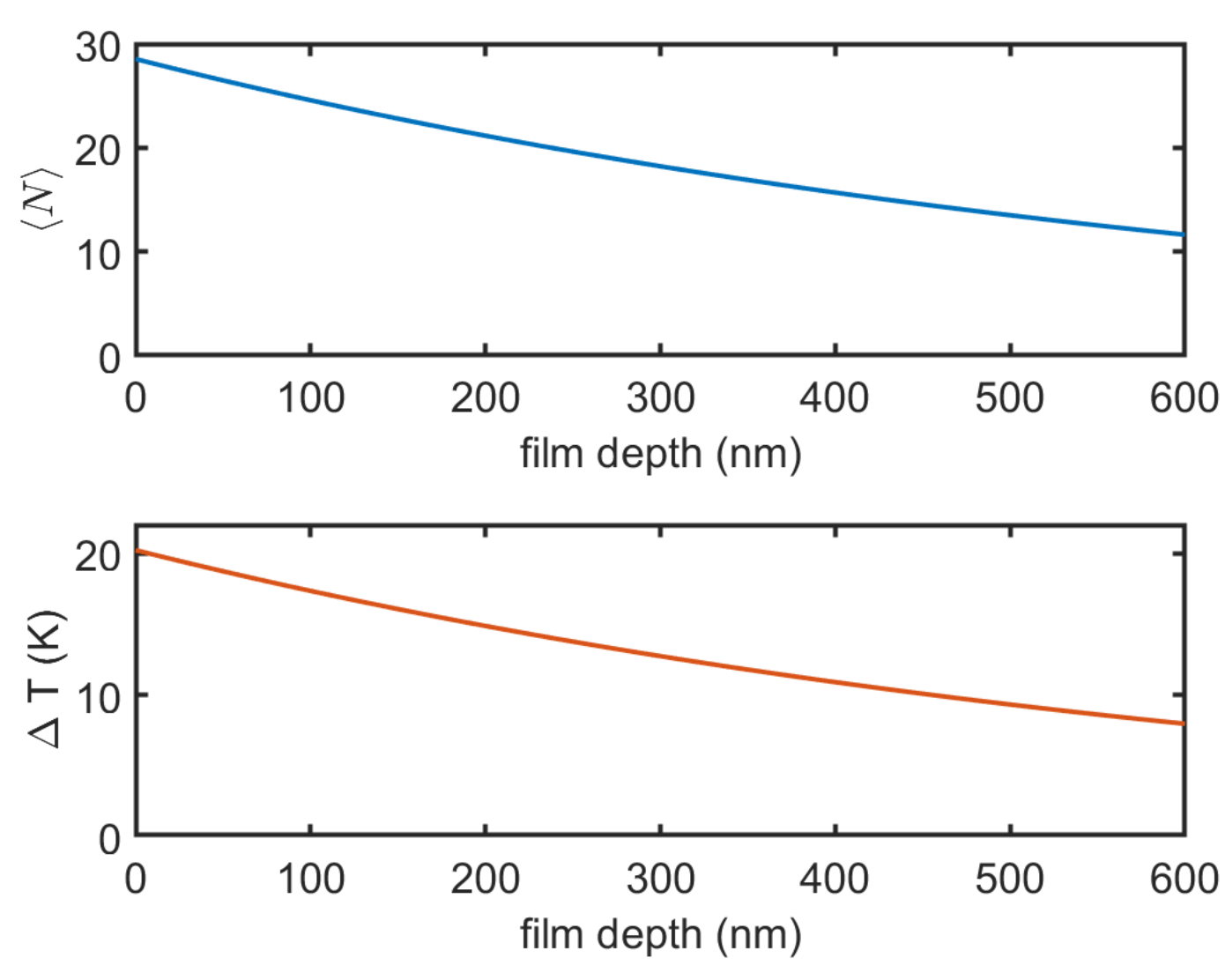

Figure S4: <N> and ΔT decay in thin-film sample. Grazing incidence experiment geometry with nearly perpendicular pump and probe beams (c.f. Figure 1) allows for more uniform excitation and probing of sample.

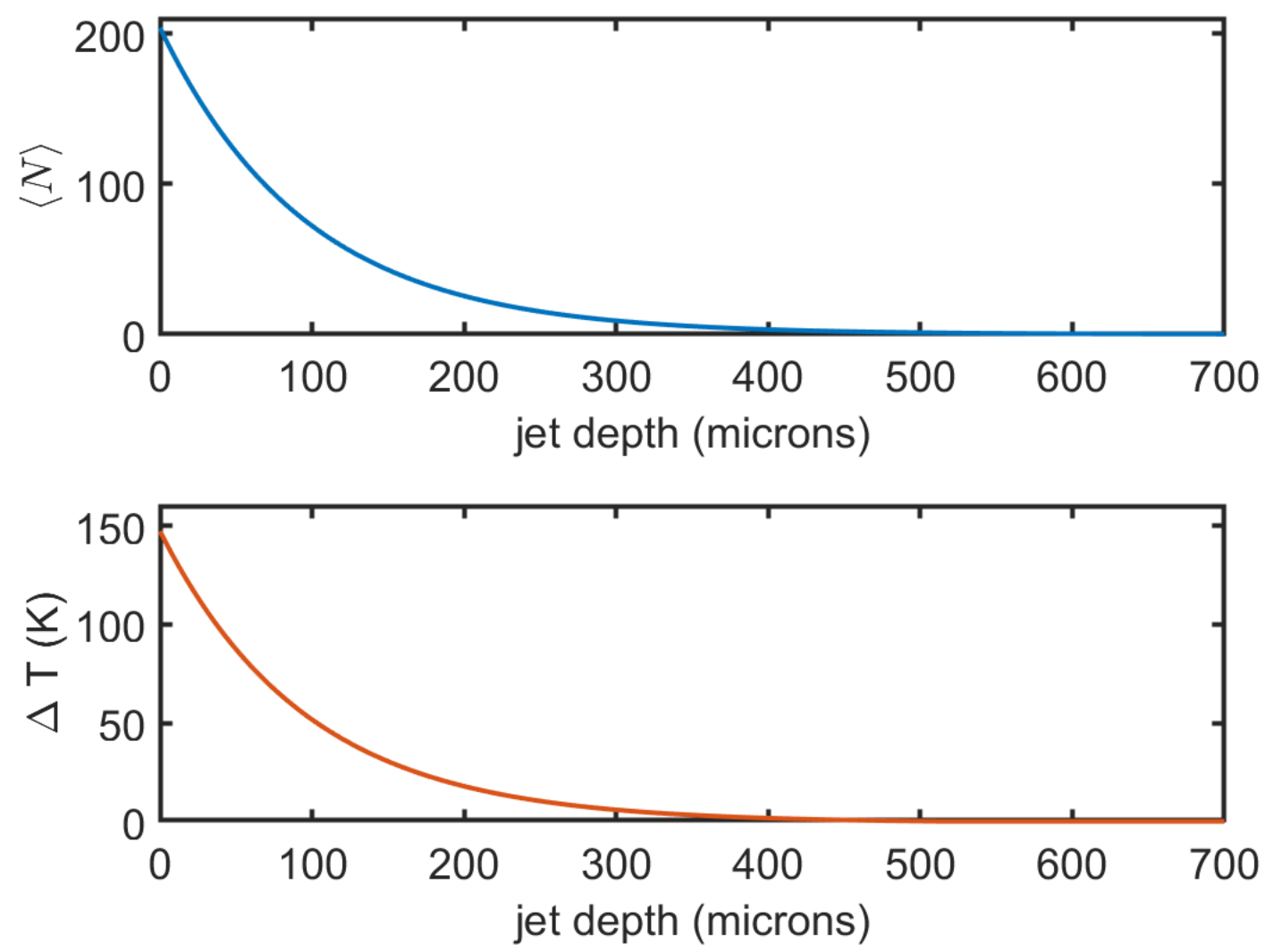

Figure S5: <N> and ΔT decay in liquid jet samples. Transmission experiment geometry with nearly parallel pump and probe beams (c.f. Figure 1) leads to attenuation of the laser as it penetrates the liquid jet, whereas the X-ray samples the jet depth evenly. This leads to less uniformity in the differential signal and potentially lower signal to noise to resolve transient heating below the sample melting point.

Inconsistency between calculated ΔT values and ΔT extracted from DWF plots

Expected average ΔT values were higher than ΔT values extracted from DWF plots for both liquid and thin-film samples, with observed experimental values 34% and 42% of depth-averaged expected values for liquid jet and thin-film setups, respectively. As discussed in the main text, the discrepancy in liquid jet data is attributed to possible nonlinear heating effects at short jet depths (See Figure S5), overestimation in the absorbed laser power, and partial cooling to surroundings prior to probing. In thin-film samples it is additionally attributed to a loss of energy to re-emission by stimulated emission. A second ASE dataset, collected from a different spot on the film than the region probed in Figure 2, is shown in Figure S6. These data demonstrate that ASE occurs well below the experimental fluence of 0.7 mJ/cm^2. Additionally, Figure S6 demonstrated the ASE increases in intensity up to and above the experimental fluence.

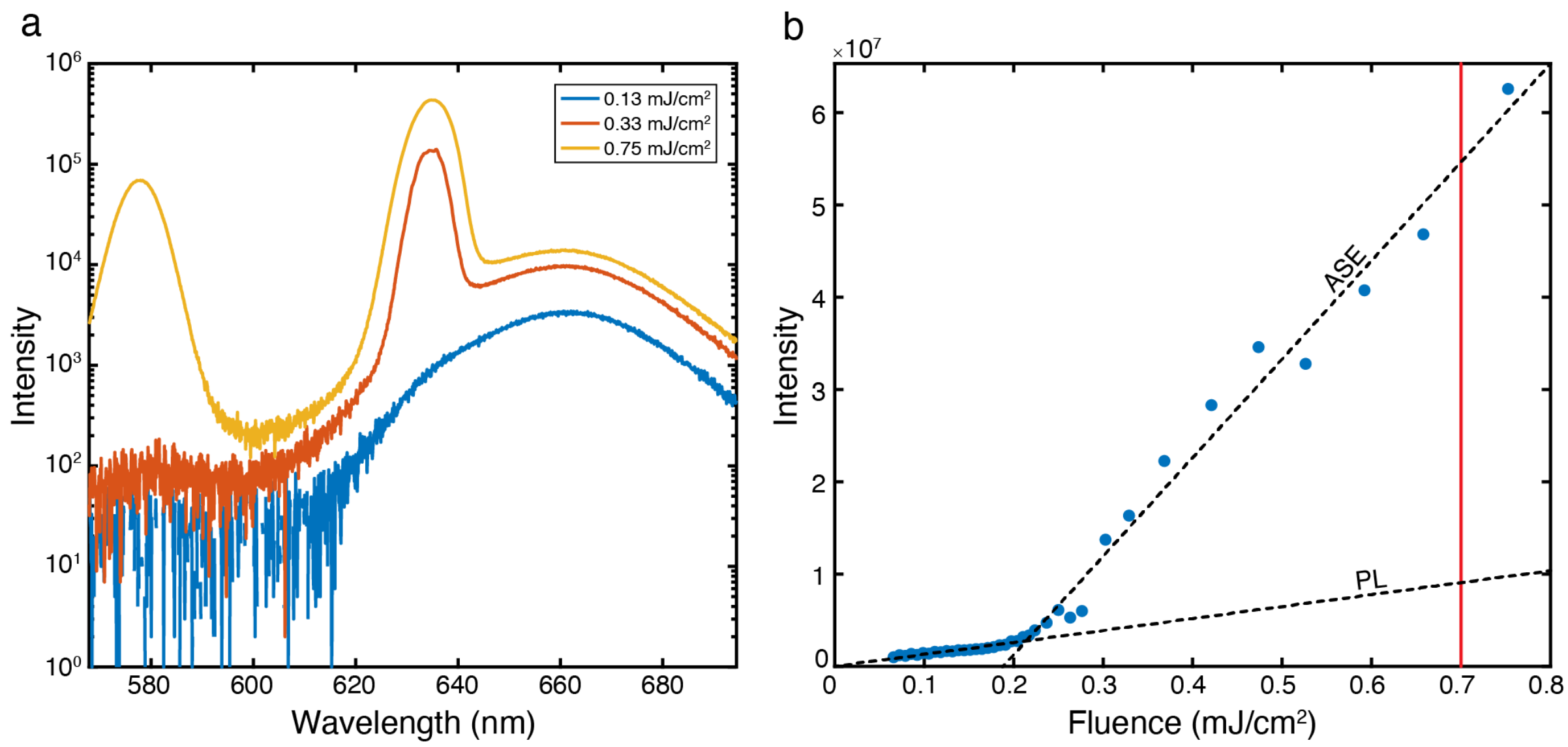

Figure S6: ASE data at a second spot in the thin-film to complement data in Figure 2. **(a)** Spectra from variable stripe length experiments, showing emission above (yellow, orange) and below (blue) ASE threshold fluence. At higher fluences, an additional ASE mode is observed, indicated by an additional peak at ~580 nm. **(b)** Integrated intensity versus fluence for all recorded fluences in VSL experiments. Data was fit to determine an ASE threshold of approximately 0.214 mJ/cm^2 at the selected spot in the film, well below the fluence used in TRXRD experiments (0.7 mJ/ cm^2), shown as a red vertical line.

F. ASE Measurement Methods

A Ti:Sapphire laser (2 kHz repetition rate, 35 fs pulse width) at Argonne National Laboratory's Center for Nanoscale Materials was used to pump the CdSe:CdS thin-film to determine the ASE threshold. The frequency doubled output at 400 nm was cylindrically focused onto the thin-film to form a 3.8 mm x 100 µm excitation stripe. Beam size was determined using a knife edge scan. Photoluminescence was collected perpendicular to the pump beam, parallel to the sample substrate. The incident power was varied using variable neutral density filters to scale incident fluence on the sample.

G. Estimation of thin-film packing density and heat capacity

The nanocrystal thin-film is comprised of CdSe:CdS nanocrystals capped with oleic acid ligands self-assembled onto a fused silica substrate. The nanocrystals are 9.4 nm in diameter with a 3.5 nm CdSe core, with the remainder being their CdS shell[2]. We follow the prior work by Ong et. al.[5] to estimate the effective packing density and heat capacity of the film. Briefly, the effective unit cell volume of the nanocrystal array is calculated assuming face-centered cubic packing as described by Minnich[6], assuming a 1.5 nm length for each oleic acid ligand[5]. With the 9.4 nm particles this gives a face diagonal distance of 21.8 nm^3

$$F_{\text{dist}} = 2d_{\text{particle}} + 2L = 2 \times 9.4 + 2 \times 1.5 = 21.8\,nm$$

and an effective unit cell volume of 916.1 nm.

$$V_{\text{eff}} = \frac{(F_{\text{dist}} \cdot \text{Cos}(45))^3}{4} = \frac{(21.8 \times 0.7071)^3}{4} = 916.1\,nm^3$$

The volume of one 9.4 nm nanocrystal is 434.9 nm^3, resulting in a volume fraction of 0.475 of the unit cell. Then the density of a single nanocrystal was calculated to be 4877 kg/m^3 from a mass-weighted average of the bulk densities of CdSe (5820 kg/m^3) and CdS (4826 kg/m^3). This yields a final film density of 2786 kg/m^3, assuming a ligand density of 895 kg/m^3 for oleic acid[5].

$$\rho_{\text{film}} = \phi \cdot \rho_{\text{particle}} + (1 - \phi) \cdot \rho_{\text{ligand}} = 2786\ kg/m^3$$

Next the thin-film heat capacity was estimated. Ong et. al. showed that a nanocrystal thin-film heat capacity is well estimated by a mass fraction weighted average of the bulk heat capacity of ligand and nanocrystal.

$$C_p = \chi_{mass,\text{particle}}\, C_{\text{particle}} + \chi_{mass,\text{ligand}}\, C_{\text{ligand}}$$

We calculate the heat capacity of the particle (C_{particle}) to be 323.5 J/Kg*K using a mass weight average of the bulk heat capacities of CdSe and CdS. The heat capacity of oleic acid is 2043 J/Kg*K.[5] Therefore, after converting the volume fraction to mass fraction for ligand and particle, the overall heat capacity of the thin-film is computed to be approximately 613.4 J/kg K.

$$C_p = \chi_{mass,\text{particle}}\, C_{\text{particle}} + \chi_{mass,\text{ligand}}\, C_{\text{ligand}} = 613.4\ J/kg \cdot K$$

H. <u>Analytical model and FEM model fitting of recovery data</u>

A finite element method (FEM) was performed to solve the transient heat transfer partial differential equation (PDE) to estimate the thermal conductivity, κ, of CdSe thin-films of various thicknesses. The PDE reads

$$\frac{\partial T_n(x,t)}{\partial t} = \alpha \nabla^2 T_n(x,t)$$

where $T_n(x,t) = \frac{T(x,t) - T_\infty}{T(x=0,t=0) - T_\infty}$, is the reduced temperature in the QD and the substrate at depth x at time t normalized over the temperature difference between the initial temperature at the top of the thin-film $T(x=0, t=0)$, and the ambient temperature T_∞, α is the thermal diffusivity, $\alpha = \frac{\kappa}{\rho C_p}$ where κ is the thermal conductivity, which will be estimated to find the best fit for the experimental temperature data, ρ is the density of the solids, and C_p is the specific heat capacity. For the silica substrate, the above values are 1.38 W m^{-1} K^{-1},[7] 2196 kg/m^3,[7] and 740 J/kgK.[8] For the QD thin-film, ρ and C_p are taken as 2786 kg/m^3 and 613 J/kgK from estimates discussed above. κ will be estimated to fit the experimental data. The initial temperature within 600nm from the surface presents a spatial decay obtained from our experimental measurements at t=0:

$$T_n(x,0) = \begin{cases} 18.59 \times e^{-1.693\times 10^6 x}, & x < 600nm \\ 0, & x \geq 600nm \end{cases}$$

We also assume that the bottom of the silica substrate remains at ambient temperature T_∞ and no heat exchange occurs between the top QD surface and the ambient environment.

The experimental recovery data plotted in Figure 3b are the average temperature across the QD thin-film, $T_{n,ave}(t) = \frac{\int_0^L \theta(x,t)dx}{L}$, and are further normalized by the initial measurement at 50 ns, *i.e.*, $T^*_{n,ave}(t) =$

$\frac{T_{n,ave}(t)-T_{n,ave,i}}{T_{n,ave,i}}$, where $T_{n,ave,i} = T_{n,ave}(50\ ns)$. To estimate the best fit of κ from FEM, we also normalized $T_n(x,t)$ the same way. The mesh grid size and time step are as small as 1nm and 1fs, respectively, to ensure the accuracy of the numerical solution.

In addition to the numerical FEM model, an analytical model assuming the QD thin-film to be a semi-infinite plate with a heat source at the surface at *t*=0 is also employed:

$$T_n(x,t) = \frac{1}{\kappa\sqrt{\frac{\pi t}{\alpha}}} e^{-\frac{x^2}{4\alpha t}}$$

The same above normalization is performed to present the data in Figure 3b. It is worth noting that the semi-infinite plate assumption is only valid when QD thin-film is significantly thicker than the thermal penetration depth, δ_p, characterizing the temperature decay from the surface. For a 90% temperature decay away from the surface, $\delta_p = 2.3\sqrt{\alpha t}$. Thus, as $t = 500$ ns, $\delta_p = 591$nm, which barely meets the requirement.

Sensitivity analysis and uncertainty in thermal conductivity modeling

Below we present a range of analytical and FEM fits for different kappa values over the 0.1 to 0.8 W m^{-1} K^{-1} range reported previously for semiconductor quantum dot (QD) films.[9,5,10–13]

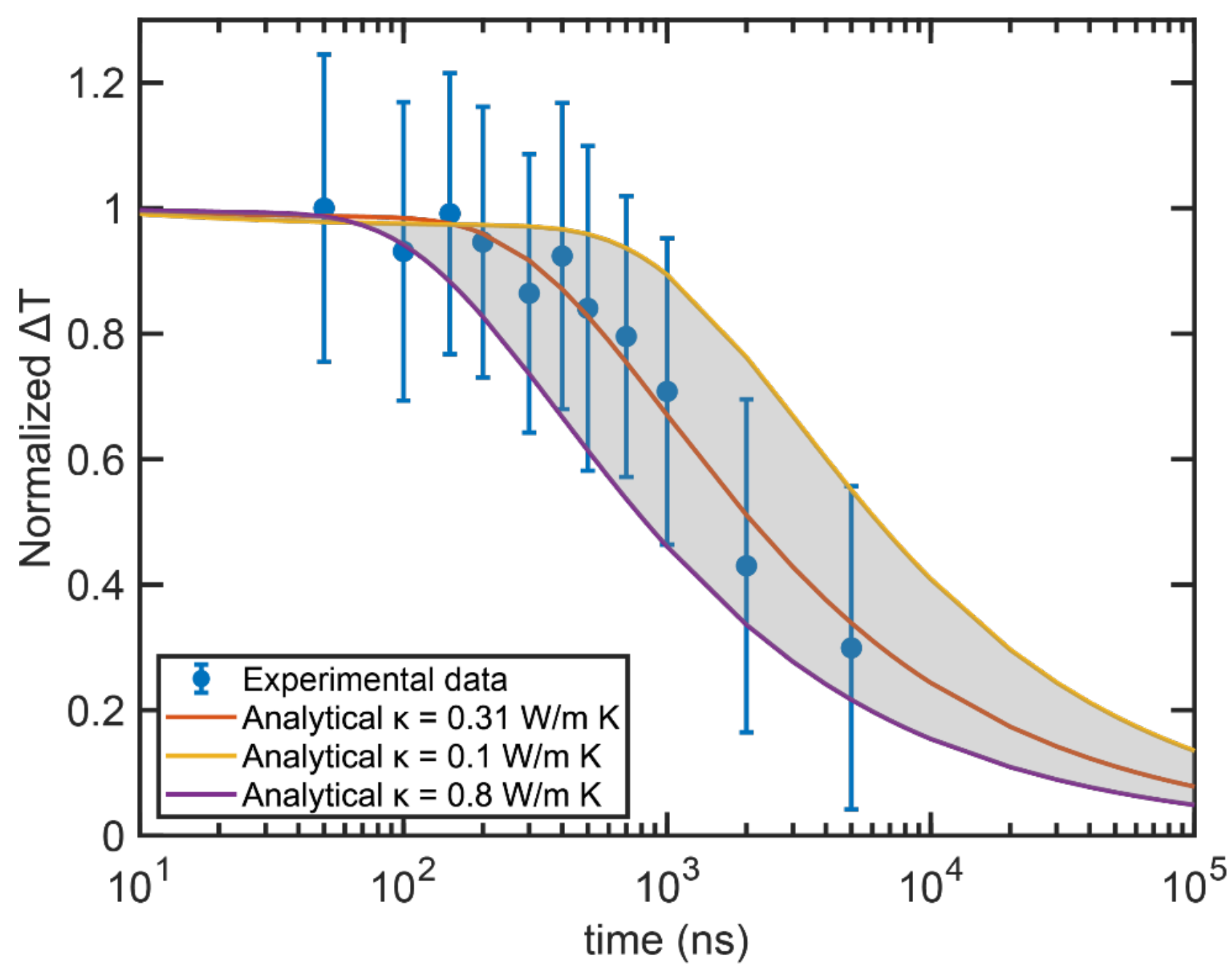

Figure S7: Thin-film recovery data plotted in blue dots and showing best fit to analytical heat transfer model in orange (κ = 0.31 W m^{-1} K^{-1}) alongside a range of reported QD thermal conductivity values (0.1-0.8 W m^{-1} K^{-1}).

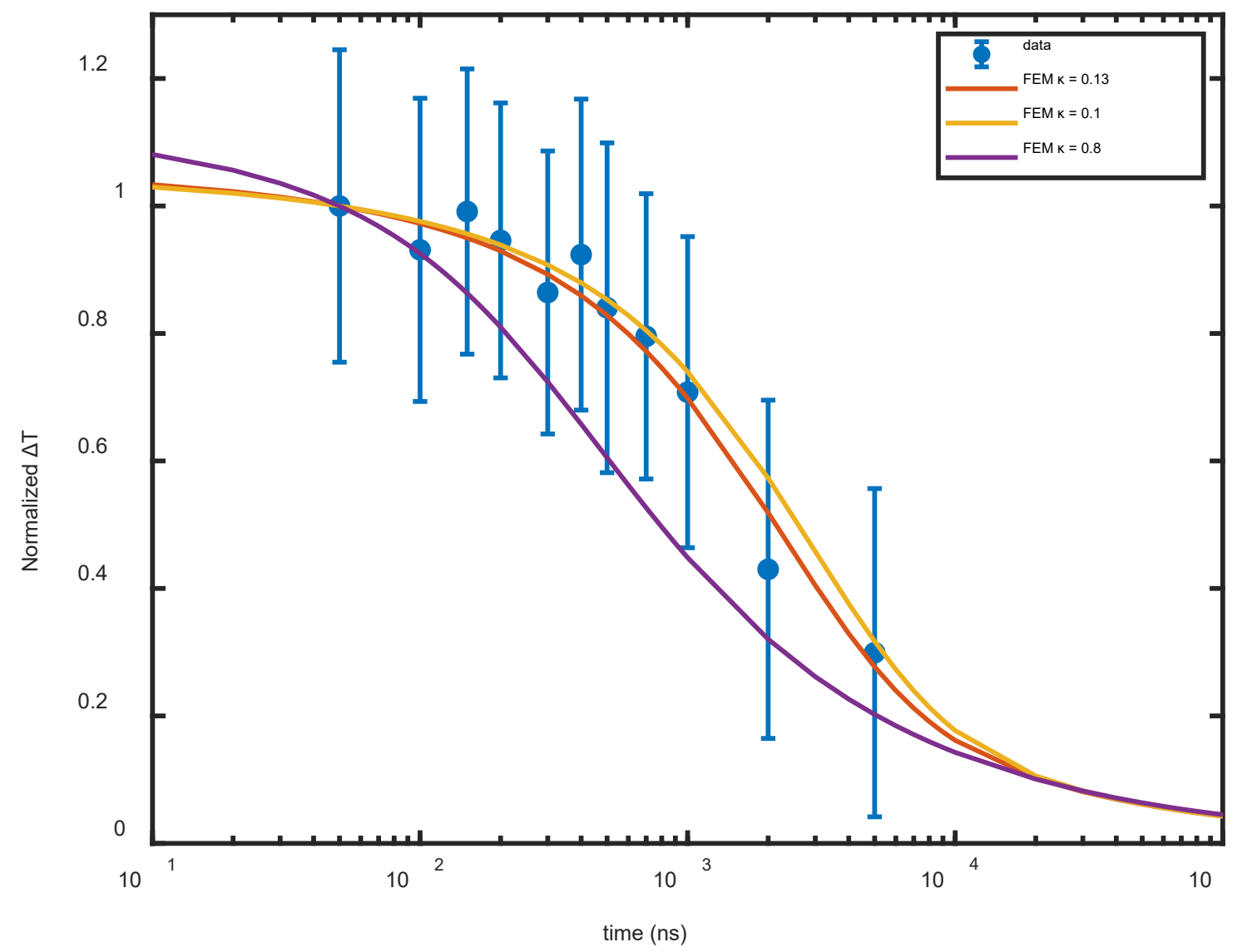

Figure S8: Thin-film recovery data plotted in blue circles with best fit with FEM model (k = 0.55 W m^{-1} K^{-1}) plotted in orange alongside a range of reported QD conductivity values (0.1-0.8 W m^{-1} K^{-1}).

Using the FEM model, decreasing the film thickness leads to faster thermal recovery (Figure S9 below). This is expected, as a thinner film presents a shorter heat diffusion path into the substrate, accelerating thermal recovery. Consequently, if the true film is thicker than modeled, whether from film thickness variations or measurement uncertainty, the model would require an artificially lower κ to reproduce the observed recovery, and the extracted κ will be underestimated. Note, that as reported in SI Section B, our thin-film film was measured to be 606 ± 68 nm using atomic force microscopy to measure step height at multiple scratches across the film, more minor than the shifts shown in Figure S9 below.

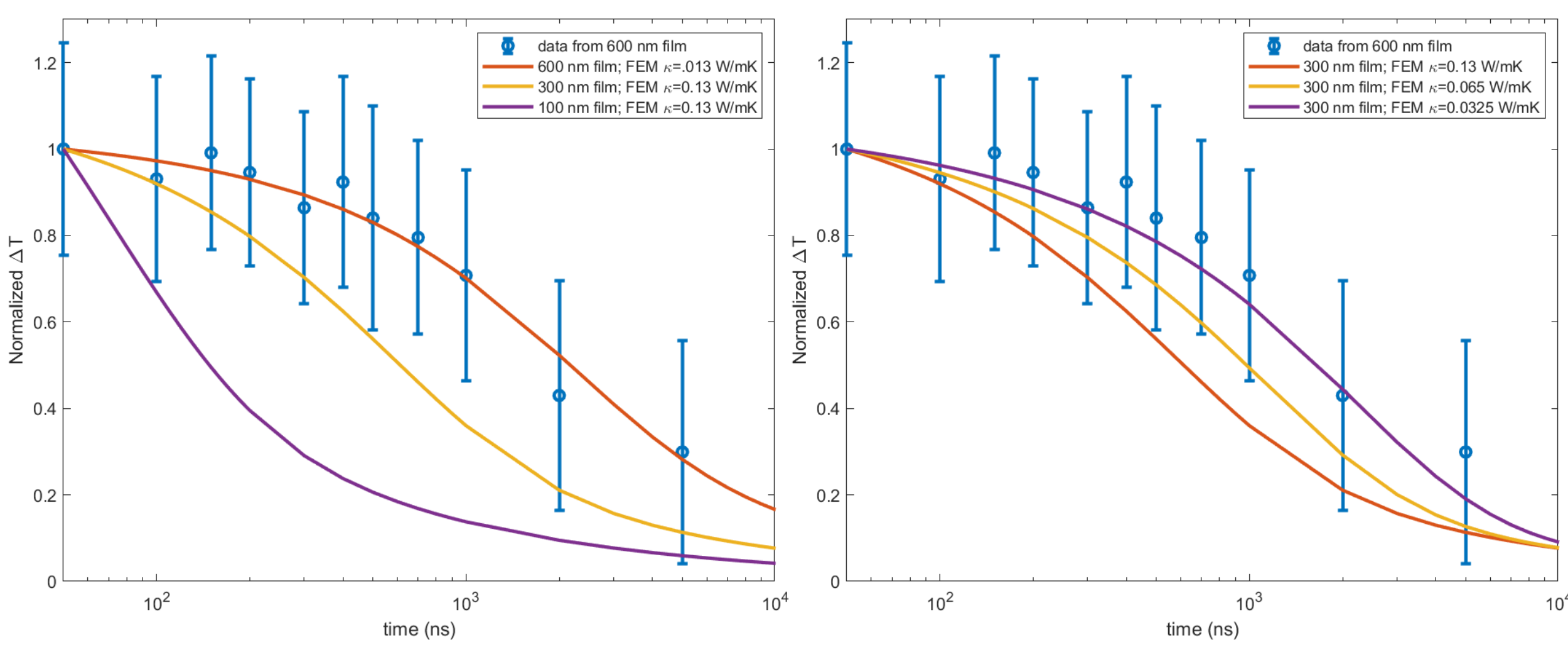

Figure S9. Demonstration of FEM model sensitivity to film thickness. (a) Solid lines show FEM simulated normalized transient temperature decay of the QD film plotted versus time with fixed thermal conductivity κ = 0.13 W m^{-1} K^{-1} for film thicknesses of 600, 300, and 100 nm. Markers show the measured normalized ΔT for the 600 nm film as a reference. Thinner films relax more rapidly owing to

the shorter heat-diffusion path to the substrate. (b) Solid lines show that an underestimation of the film thickness yields a lower thermal conductivity prediction.

Changes in absorption depth will also have an impact on the thermal recovery. As seen in Figure S10 changes from 600 to 100 nm of absorption depth using the same exponential optical absorption decay as described in the manuscript alters the decay curve. These results demonstrate that when the actual absorption depth is shorter than the QD film thickness, the predicted κ is lower than the real value.

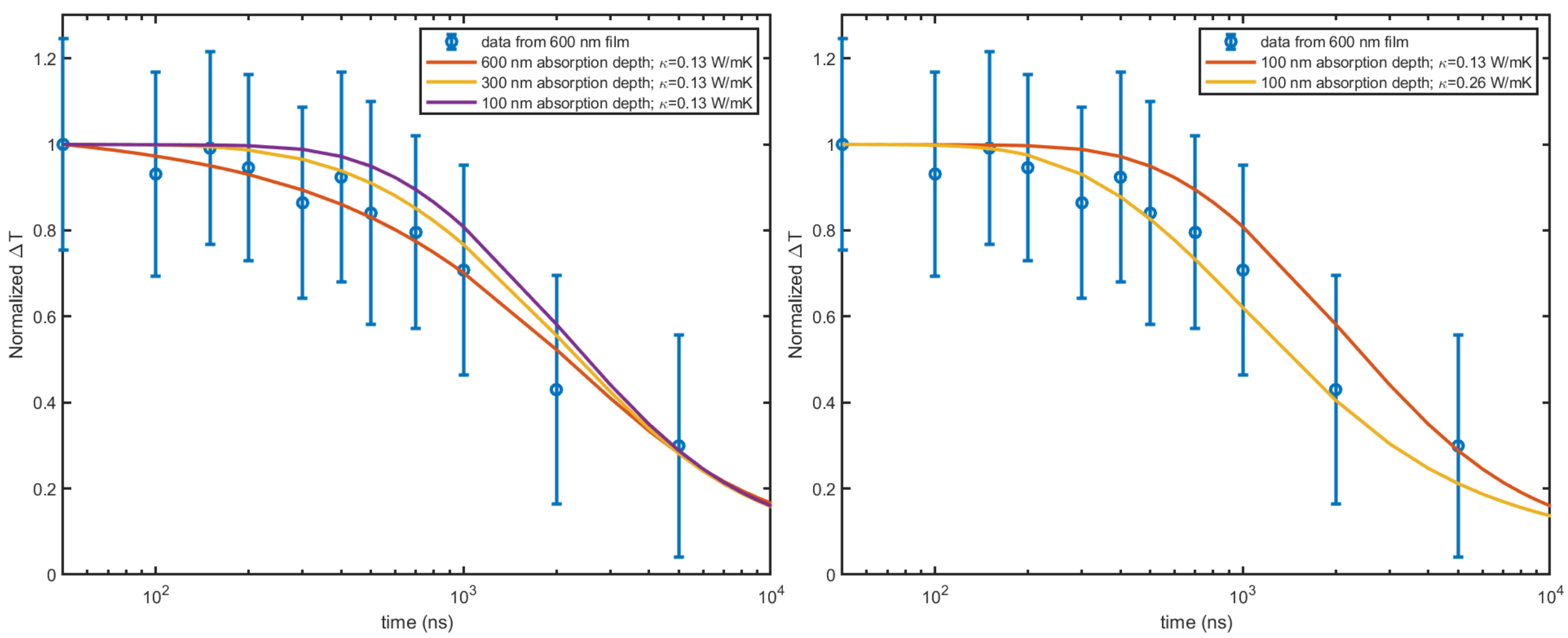

Figure S10: Demonstration of impact on FEM model as absorption depth is changed for the 600 nm film. (a) Blue markers show the measured data as a reference. FEM results are shown in colored lines for the absorption depth varying from 600nm, 300nm, to 100nm with constant κ=0.13 W^{-1} m K^{-1} for each. The changes in the normalized temperature recovery are dominated by the slower process of heat dissipation into the substrate, but are influenced by the variations in achieving quasi-thermal equilibrium within the film from changing the absorption depth. (b) Solid lines show FEM results for an imposed lower 100 nm absorption depth. Here, we demonstrate that an underestimation of the absorption depth yields a higher predicted thermal conductivity.

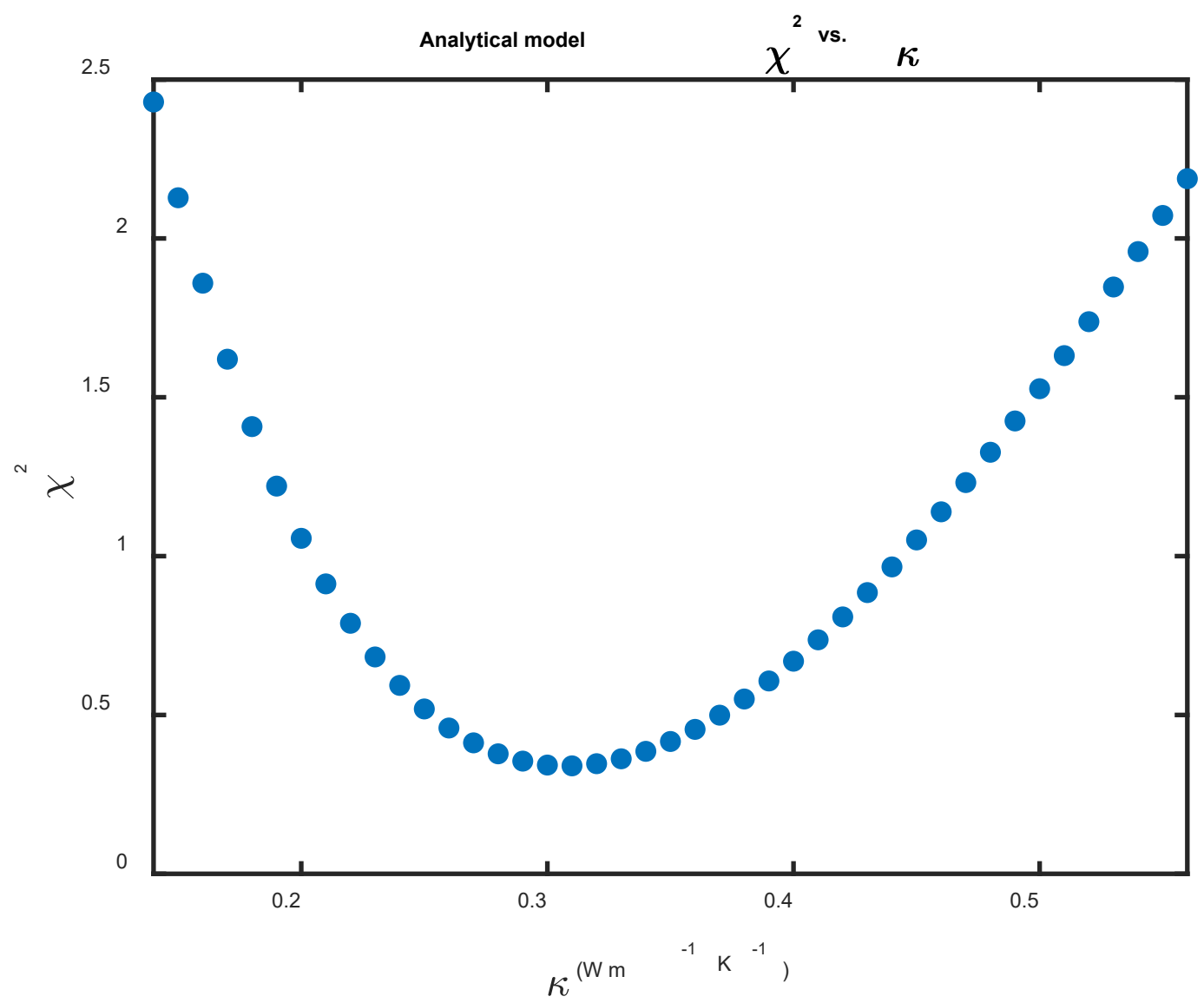

Figure S11: Chi-squared (χ^2) between the Analytical-simulated and measured temperature recovery as a function of the QD-film thermal conductivity κ. The minimum at κ = 0.31 W m⁻¹ K⁻¹ defines the best-fit value. Using the standard one-parameter criterion $\Delta\chi^2$= 1, we obtain an uncertainty range of κ from 0.18 to 0.48 W m⁻¹ K⁻¹ or κ = $0.31^{+0.17}_{-0.13}$ W m⁻¹ K⁻¹.

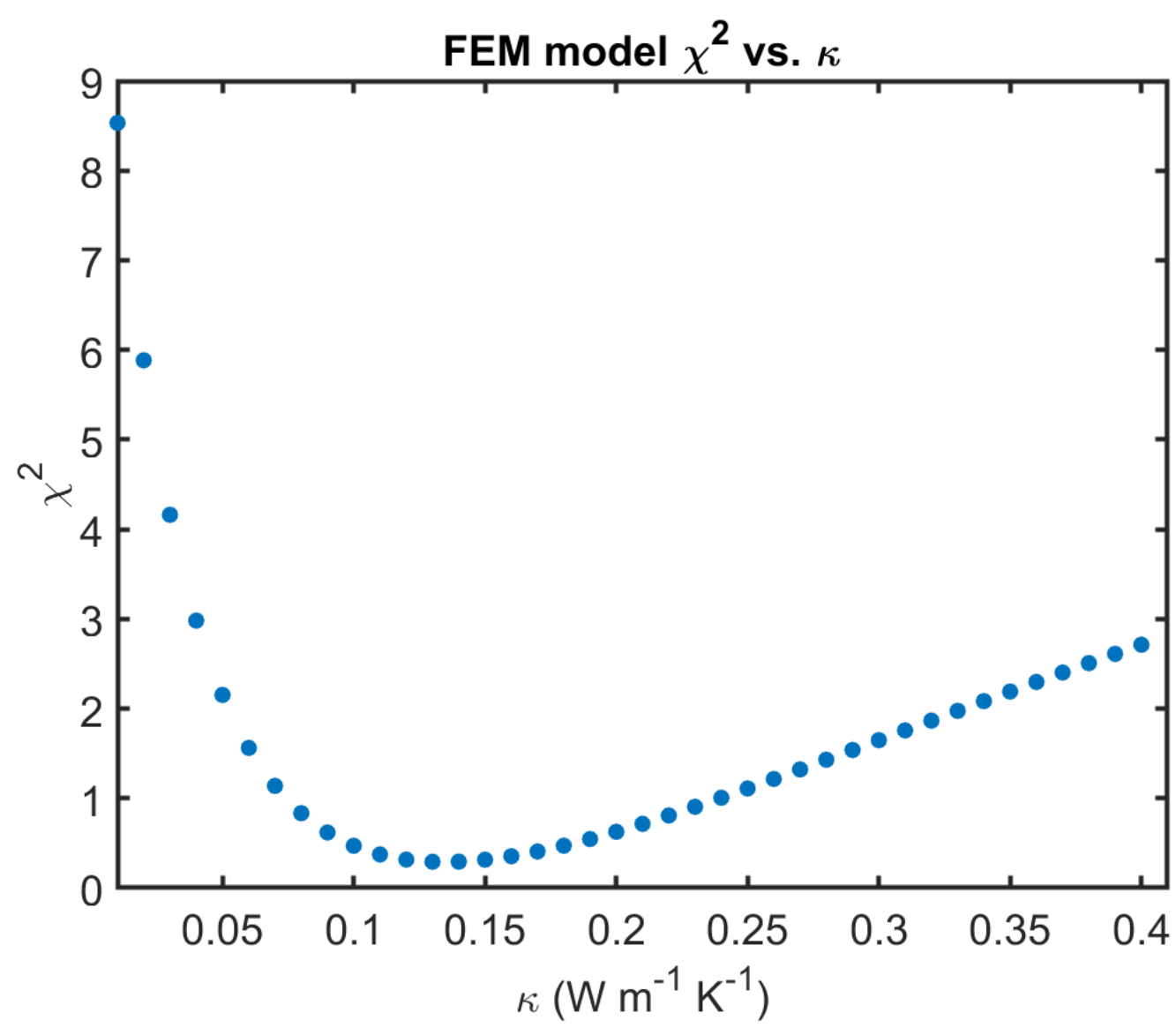

Figure S12: Chi-squared (χ^2) between the FEM-simulated and measured temperature recovery as a function of the QD-film thermal conductivity κ. The minimum at κ = 0.13 W m⁻¹ K⁻¹ defines the best-fit value. Using the standard one-parameter criterion $\Delta\chi^2$= 1, we obtain an uncertainty range of κ from 0.06 to 0.27 W m⁻¹ K⁻¹ or κ = $0.13^{+0.14}_{-0.07}$ W m⁻¹ K⁻¹.

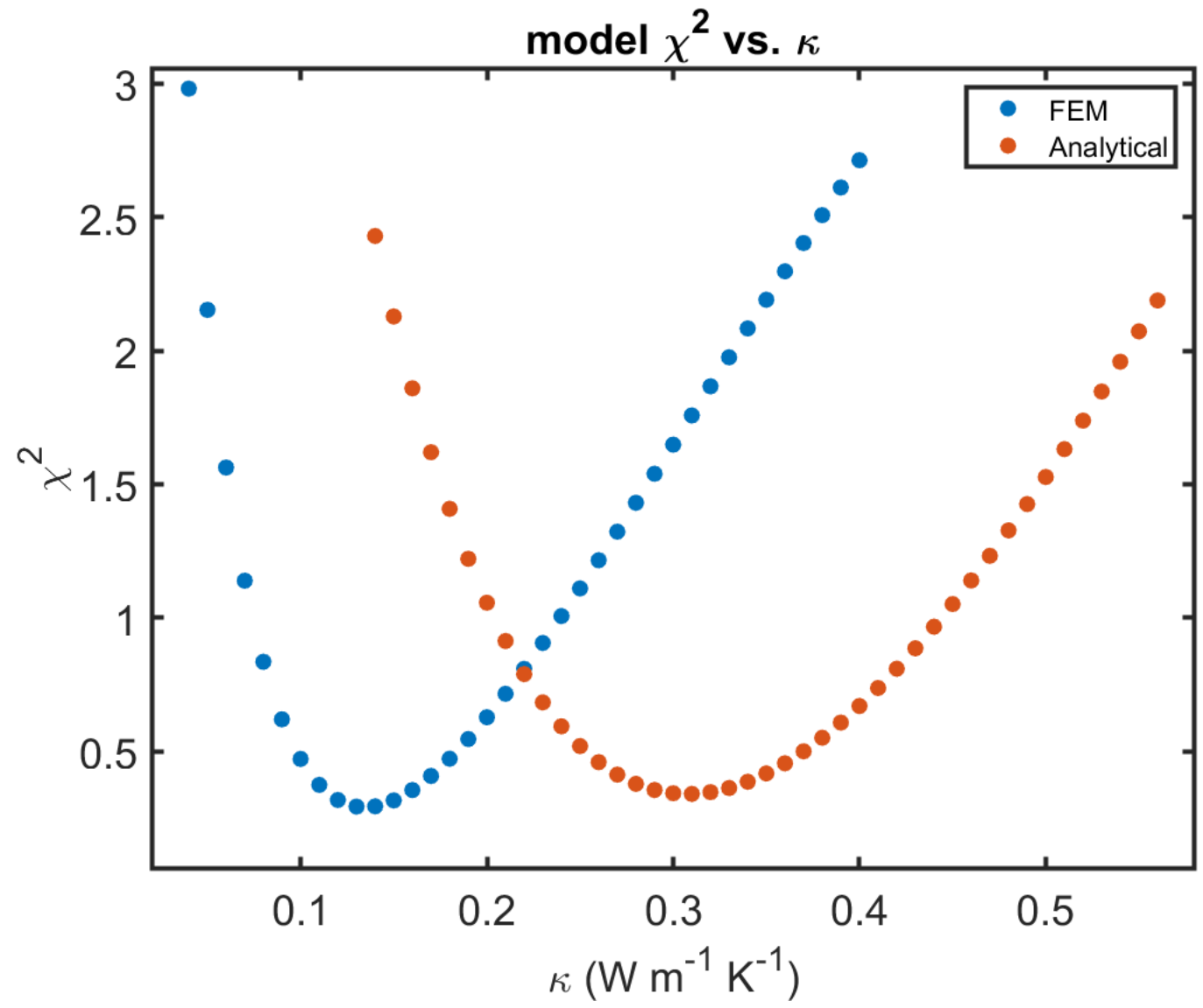

Figure S13: Comparison of the chi-squared (χ^2) goodness of fit measured temperature recovery and that simulated by FEM and analytical models, as a function of the QD-film thermal conductivity κ. The FEM simulation returns a best fit kappa of $\kappa = 0.13^{+0.14}_{-0.07}$ W m^{-1} K^{-1}, and the analytical model returns a $\kappa = 0.31^{+0.17}_{-0.13}$ W m^{-1} K^{-1}. The offset between the best-fit values reflects simplistic assumption in the analytical model, nevertheless, the values agree within their respective uncertainties.

I. Application of FEM model to approximate CW heating

Employing our FEM model, we adjust the heating parameters to shift from an impulsive ultrafast excitation to estimating the steady heating of our QD thin-film under a 400 nm continuous-wave (CW) pump. For QD active layers pumped by CW lasers in state-of-the-art devices, typical fluences are on the order of 10 kW/cm^2.[14,15] We therefore use this value and assume 40% of the pump is absorbed in the film based on UV–Vis absorption data, corresponding to an absorbed power of 4 kW/cm^2. Dividing by the film thickness yields a volumetric power density of $6.7 \times\ 10^{13}\ W\ m^{-3}$ absorbed the sample. As seen in Figure S14 the film climbs from an initial temperature of 300 K to 400 K within 5 µs.

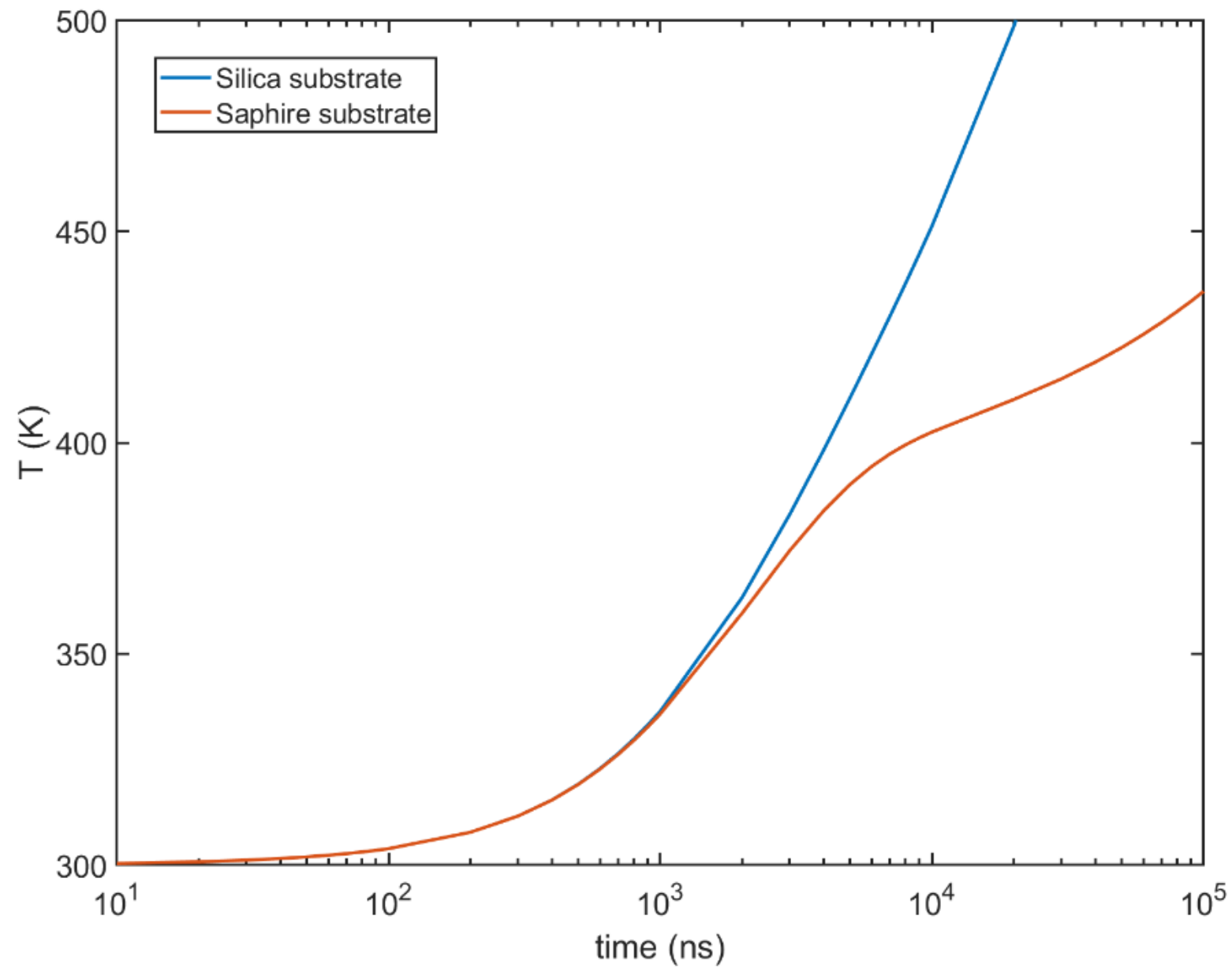

Figure S14: Simulation of film temperature over time under continuous-wave excitation at 10 kW/cm² assuming 60% absorption. The film temperature rises by 100 K within 5 µs on a silica substrate. Moving to a sapphire substrate partially mitigates the temperature rise so that the film rises by only 90 K within the first 5 µs, and the heating slows considerably beyond 100 µs. However, given the quantum dots' high absorption coefficient at 400 nm, the film thickness, and the film's low thermal conductivity, CW pumping will lead to rapid temperature rise and associated performance degradation without dedicated thermal transport and heat sink engineering.

Supporting Information References

(1) Guzelturk, B.; Cotts, B. L.; Jasrasaria, D.; Philbin, J. P.; Hanifi, D. A.; Koscher, B. A.; Balan, A. D.; Curling, E.; Zajac, M.; Park, S.; Yazdani, N.; Nyby, C.; Kamysbayev, V.; Fischer, S.; Nett, Z.; Shen, X.; Kozina, M. E.; Lin, M.-F.; Reid, A. H.; Weathersby, S. P.; Schaller, R. D.; Wood, V.; Wang, X.; Dionne, J. A.; Talapin, D. V.; Alivisatos, A. P.; Salleo, A.; Rabani, E.; Lindenberg, A. M. Dynamic Lattice Distortions Driven by Surface Trapping in Semiconductor Nanocrystals. *Nat. Commun.* **2021**, *12* (1), 1860. https://doi.org/10.1038/s41467-021-22116-0.

(2) Hanifi, D. A.; Bronstein, N. D.; Koscher, B. A.; Nett, Z.; Swabeck, J. K.; Takano, K.; Schwartzberg, A. M.; Maserati, L.; Vandewal, K.; van de Burgt, Y.; Salleo, A.; Alivisatos, A. P. Redefining Near-Unity Luminescence in Quantum Dots with Photothermal Threshold Quantum Yield. *Science* **2019**, *363* (6432), 1199–1202. https://doi.org/10.1126/science.aat3803.

(3) Gates-Rector, S.; Blanton, T. The Powder Diffraction File: A Quality Materials Characterization Database. *Powder Diffr.* **2019**, *34* (4), 352–360. https://doi.org/10.1017/S0885715619000812.

(4) Moses Badlyan, N.; Biermann, A.; Aubert, T.; Hens, Z.; Maultzsch, J. Thermal Expansion of Colloidal CdSe/CdS Core/Shell Quantum Dots. *Phys. Rev. B* **2019**, *99* (19), 195425. https://doi.org/10.1103/PhysRevB.99.195425.

(5) Ong, W.-L.; Rupich, S. M.; Talapin, D. V.; McGaughey, A. J. H.; Malen, J. A. Surface Chemistry Mediates Thermal Transport in Three-Dimensional Nanocrystal Arrays. *Nat. Mater.* **2013**, *12* (5), 410–415. https://doi.org/10.1038/nmat3596.

(6) Minnich, A.; Chen, G. Modified Effective Medium Formulation for the Thermal Conductivity of Nanocomposites. *Appl. Phys. Lett.* **2007**, *91* (7), 073105. https://doi.org/10.1063/1.2771040.

(7) *CRC Handbook of Chemistry and Physics*, 97th ed.; Haynes, W. M., Ed.; CRC Press: Boca Raton, 2016. https://doi.org/10.1201/9781315380476.
(8) *Fused Silica | SiO2 Material Properties*. https://accuratus.com/fused.html (accessed 2024-10-02).
(9) Urban, J. J. Prospects for Thermoelectricity in Quantum Dot Hybrid Arrays. *Nat. Nanotechnol.* **2015**, *10* (12), 997–1001. https://doi.org/10.1038/nnano.2015.289.
(10) Feser, J. P.; Chan, E. M.; Majumdar, A.; Segalman, R. A.; Urban, J. J. Ultralow Thermal Conductivity in Polycrystalline CdSe Thin Films with Controlled Grain Size. *Nano Lett.* **2013**, *13* (5), 2122–2127. https://doi.org/10.1021/nl400531f.
(11) Wang, Z.; Singaravelu, A. S. S.; Dai, R.; Nian, Q.; Chawla, N.; Wang, R. Y. Ligand Crosslinking Boosts Thermal Transport in Colloidal Nanocrystal Solids. *Angew. Chem. Int. Ed.* **2020**, *59* (24), 9556–9563. https://doi.org/10.1002/anie.201916760.
(12) Wang, Z.; Christodoulides, A. D.; Dai, L.; Zhou, Y.; Dai, R.; Xu, Y.; Nian, Q.; Wang, J.; Malen, J. A.; Wang, R. Y. Nanocrystal Ordering Enhances Thermal Transport and Mechanics in Single-Domain Colloidal Nanocrystal Superlattices. *Nano Lett.* **2022**, *22* (12), 4669–4676. https://doi.org/10.1021/acs.nanolett.2c00544.
(13) Park, J. Y.; Chen, D.; Li, S.; Xia, Y.; Diroll, B. T.; Guo, P. Quantifying Size Effects on Thermal Transport in CsPbBr3 Nanocrystal Films. *Nano Lett.* **2025**, *25* (39), 14286–14292. https://doi.org/10.1021/acs.nanolett.5c03215.
(14) Adachi, M. M.; Fan, F.; Sellan, D. P.; Hoogland, S.; Voznyy, O.; Houtepen, A. J.; Parrish, K. D.; Kanjanaboos, P.; Malen, J. A.; Sargent, E. H. Microsecond-Sustained Lasing from Colloidal Quantum Dot Solids. *Nat. Commun.* **2015**, *6* (1), 8694. https://doi.org/10.1038/ncomms9694.
(15) Hahm, D.; Chang, J. H.; Jeong, B. G.; Park, P.; Kim, J.; Lee, S.; Choi, J.; Kim, W. D.; Rhee, S.; Lim, J.; Lee, D. C.; Lee, C.; Char, K.; Bae, W. K. Design Principle for Bright, Robust, and Color-Pure InP/$ZnSe_xS_{1-x}$/ZnS Heterostructures. *Chem. Mater.* **2019**, *31* (9), 3476–3484. https://doi.org/10.1021/acs.chemmater.9b00740.
